# Impact of COVID-19 on Older Employees of a Large State University: Findings From a Mixed-Methods Study

**DOI:** 10.1093/geroni/igab046.247

**Published:** 2021-12-17

**Authors:** Zhao Chen, Amanda Sokan

## Abstract

Burgeoning research on the effects of COVID-19 and university experiences in the U.S. tends to focus on transmission of COVID-19 virus or student-related consequences of COVID-19. However, none to our knowledge examine the effects on older university employees. Universities employ a higher percentage of older adults with diverse job responsibilities and socioeconomic status, presenting a unique closed community for understanding the pandemic’s consequences for older adults. Our aims are to: 1) understand older university employees’ concerns related to COVID-19, 2) develop intervention strategies to mitigate the adverse impact of the COVID-19 pandemic on the health and wellbeing of older employees, and 3) test the interventions within the target population to help reduce stress and promote wellbeing. Using a community participatory approach, we sought input from employees aged 50 and older at the University of Arizona. Mixed methods were used to collect qualitative (six focus groups; N= 24) and quantitative (online survey; N=1030) data. We conducted and evaluated a set of interventions (i.e., virtual Tai Chi and Qigong, walking exercises, and meditation) using focus group feedback, process evaluation, and outcome assessment with validated questionnaires on sleep quality, mindfulness and psychological wellbeing. Findings show that a significant percentage of older employees worried about getting COVID-19 and had experienced undesirable changes in sleep quality, weight, and physical activity, and concerns about caregiving; however, we also observed psychological resilience in this population. The study highlights the importance of developing immediate and effective programs for promoting health and wellbeing for older employees during the pandemic.

